# Evaluating Prostate-Specific Antigen (PSA) Density Thresholds for Detecting Clinically Significant Prostate Cancer in Prostate Imaging Reporting and Data System (PI-RADS) 3 Lesions: A Retrospective Cohort Study

**DOI:** 10.7759/cureus.96432

**Published:** 2025-11-09

**Authors:** Muhammad A Nazir, Megan Scobie, Vignesh Balasubaramaniam, Anurag Agarwal, Ashok Kailasa, Shanmugasigamani Kannan, Kyriacos Alexandrou, Mohanarangam Thangavelu, Krassen Donev

**Affiliations:** 1 General Surgery, Wrexham Maelor Hospital, Wrexham, GBR; 2 Surgery, Betsi Cadwaladr University Health Board, Bangor, GBR; 3 Urology, Betsi Cadwaladr University Health Board, Bangor, GBR

**Keywords:** clinically significant prostate cancer, multiparametric mri, pi-rads 3, prostate-specific antigen density, psad

## Abstract

Introduction

Prostate cancer is one of the most common cancers in the world. Equivocal prostate magnetic resonance imaging (MRI) findings (Prostate Imaging Reporting and Data System [PI-RADS] 3) remain a diagnostic challenge. The use of prostate-specific antigen density (PSAD) has been suggested to help risk-stratify this cohort of patients to help guide the decision to biopsy. This study aimed to evaluate the diagnostic performance of PSAD across multiple cut-offs in detecting clinically significant prostate cancer (csPCa) in patients with PI-RADS 3 lesions.

Methods

A retrospective audit of 194 men with PI-RADS 3 lesions on multiparametric magnetic resonance imaging (mpMRI) who underwent prostate biopsy was performed. Age, prostate-specific antigen (PSA), prostate volume, and PSAD were collected. Receiver operating characteristic (ROC) analysis was used to assess model accuracy. Univariate and multivariate logistic regression were performed to identify significant predictors of csPCa. Sensitivity, specificity, positive predictive value (PPV), and negative predictive value (NPV) were calculated at PSAD thresholds of 0.10, 0.15, and 0.20 ng/mL^2^.

Results

csPCa was identified in 47 (24.2%) of patients. Median prostate volume was significantly lower, and PSAD was considerably higher, in this cohort of patients (p < 0.001). ROC analysis showed an area under the curve (AUC) of 0.72 (95% confidence interval [CI] 0.65-0.79). Multivariate analysis confirmed that PSAD remained a significant predictor for csPCa (odds ratio (OR) 1.6, 95% CI 1.2-2.0, p < 0.001). Sensitivity and specificity at PSAD ≥ 0.10 ng/mL^2^, ≥ 0.15 ng/mL^2^, and ≥0.20 ng/mL^2^ were 0.92/0.29, 0.68/0.65, and 0.53/0.84, respectively. PPV increased (0.29, 0.39, and 0.52) while NPV remained high (0.91,0.87,0.85) across these cut-offs.

Conclusion

PSAD is demonstrated as a good predictor for csPCa in PI-RADS 3 lesions. A threshold of 0.15 ng/mL^2^ provides a good balance between sensitivity and specificity and supports its use in determining biopsy decisions in this cohort. Validation in larger multicentre cohorts is warranted.

## Introduction

Prostate cancer remains the second most commonly diagnosed cancer worldwide [1]. The current inevitable demographic shift in our ageing population brings about a higher burden of prostate cancer. It is predicted that by 2040, the burden of prostate cancer will affect 2.4 million people in that year alone [2].

The diagnostic pathway for prostate cancer has evolved over the last decade. Prostate multiparametric MRI (mpMRI) is now the primary investigation, replacing transrectal ultrasound (TRUS)-guided biopsy, which was used historically [3]. The PROMIS study has been pivotal in the development of diagnosing clinically significant prostate cancers (csPCa). Using mpMRI, 18% more csPCa were detected, whilst 27% were able to omit a biopsy based on MRI findings [4]. 

Despite this progress in detecting csPCa and reducing unnecessary biopsies, papers have raised concerns about interobserver variability in mpMRI reporting. The Prostate Imaging Reporting and Data System (PI-RADS) v2.1, jointly developed by the American College of Radiology (ACR), the European Society of Urogenital Radiology (ESUR), and the AdMeTech Foundation as an open-access tool to standardize prostate MRI interpretation and reporting, was introduced to address this [5,6]. It is a five-point scoring system that aims to standardise interpretation and classify patients according to the likelihood of having csPCa, defined as a Gleason score of 7 or higher. The Gleason score is a histological grading system that evaluates prostate cancer to indicate tumour differentiation and aggressiveness [7]. In PI-RADS 3, the chance of having a csPCa is equivocal [8]. Previous systematic reviews have reported detection rates of approximately 13-20% in patients with csPCa and having PI-RADS 3 on MRI [9,10].

The European Association of Urology (EAU) provides guidelines on further investigation and management of prostate cancer based on the PI-RADS score and prostate-specific antigen density (PSAD). The recommendations for PI-RADS 3 are based upon a study by Schoots et al., which uses PSAD to define risk and further stratify management [11]. Low risk PSAD is defined as <0.10 ng/mL2; intermediate/low risk is 0.10-0.15 ng/mL2; intermediate/high risk is 0.15-0.20 ng/mL2; and high risk is >0.2 ng/mL2. The management for each, respectively, is no biopsy, consider biopsy, highly consider biopsy, and biopsy required [11,12]. This study aims to evaluate multiple PSAD cut-off values in patients with PI-RADS 3 lesions on mpMRI, assessing their diagnostic performance for csPCa and their potential role in optimising biopsy decision-making.

## Materials and methods

Study design

This is a retrospective, single-centre cohort study conducted at a district general hospital in the United Kingdom. This study was registered as an audit at Ysbyty Gwynedd Hospital, with no alteration to the standard of patient care and clinical pathway. Inclusion criteria comprised patients with PI-RADS 3 findings on mpMRI and no previous diagnosis of prostate cancer who underwent local anaesthetic transperineal biopsy (LATP) between January 1, 2022, and June 30, 2025. Exclusion criteria included patients with missing histology results, prostate-specific antigen (PSA) values, prostate volume, or a documented PI-RADS score on MRI. A total of 194 patients’ data was analysed.

Data collection

Clinical, radiological, and histopathological data were extracted using the hospital’s electronic health records, multidisciplinary outcome sheets, and outpatient clinic letters. The variables included age (years), pre-MRI serum PSA (ng/mL), prostate volume (mL) as reported on the MRI, and PI-RADS score from the MRI report. PSAD is calculated as PSA divided by prostate volume. Histology was recorded for each patient and graded using the Gleason score. Two Consultant Urologists carried out the prostate biopsies, with a minimum of 24 core systematic biopsies obtained as per local guidelines, and various Consultant Radiologists reported the MRI findings. Data were entered into a secure Microsoft Excel spreadsheet (Redmond, WA, USA). 

Outcome definition

csPCa was defined as those having a Gleason Score of ≥ 7, and non-csPCa was defined as those with a Gleason score of 6 or benign histology. 

Statistical analysis

Statistical analysis was performed using SPSS Statistics version 26 (IBM Corp., Armonk, NY, USA). Descriptive statistics were used to summarise the findings. Categorical data were displayed as frequency, and continuous data (Age, PSA, Prostate Volume, PSAD) were summarised as medians with interquartile ranges (IQR). Differences in continuous variables between patients with and without clinically significant prostate cancer were assessed using the Mann-Whitney U test. Univariate and multivariate logistic regression models were used to evaluate variables associated with csPCa. 

Univariate analysis was done using PSA, prostate volume, PSAD, and age as the independent variables. The dependent variable was histology findings (csPCa vs. non-CS-PCa). In the multivariate analysis, PSAD and age were used as the covariates, and the dependent variable was histology findings (csPCa vs non csPCa). Only PSAD and age were used in the multivariate model to avoid multicollinearity, as PSA and prostate volume are both components of PSAD. PSAD was scaled by a factor of 10 to improve the interpretability of the odds ratio and express it per 0.1 ng/mL2 increase, providing a clinically meaningful interpretation. Effect sizes were expressed as odds ratios (ORs) with 95% confidence intervals (CIs). Receiver operating characteristic (ROC) analysis was performed to assess the discriminative performance of PSAD, with the area under the curve (AUC). Diagnostic performance was calculated for fixed thresholds (0.10, 0.15, 0.20 ng/mL2), including sensitivity, specificity, positive predictive value (PPV), and negative predictive value (NPV). Statistical significance was set at p < 0.05, and 95% CIs were calculated. As this project was designed as a retrospective audit, no sample size calculation was undertaken, and no formal hypothesis was proposed.

Ethics/data governance 

All data were anonymised and stored securely in accordance with institutional data protection policies. As this project was classified as an audit, formal ethical approval was not required. 

## Results

Patient characteristics 

A total of 194 patients met the inclusion criteria. The median age was 67 years (IQR 62-72). The median PSA was 7.3 ng/mL (IQR 5.6-9.3), and the median prostate volume was 52 mL (IQR 35-65.7). The median PSAD was 0.142 ng/mL (IQR 0.101-0.195). One case with a PSAD value of 2.825 ng/mL2 was identified as an extreme observation. This value corresponded to a patient with csPCa and represented a true clinical finding rather than a measurement error. Diagnostic checks (Cook’s distance, leverage, and standardised residuals) indicated no influence on the regression model; therefore, it was included in the final dataset. csPCa was detected in 47 (24.2%) patients. The remaining 147 (75.8%) of patients had either Gleason 6 disease or benign histology. There was no significant difference in age or total PSA between groups; however, prostate volume was significantly lower and PSAD was significantly higher among patients with csPCa (p<0.001 for both). Baseline characteristics for the individual groups are summarised in Table 1. 

**Table 1:** Baseline characteristics. *Comparisons between the groups were performed using the Mann-Whitney U test. N/A: Not Applicable, PSA: Prostate Specific Antigen, csPCa: Clinically Significant Prostate Cancer, IQR: Interquartile Range

Clinical Characteristics	Total Patients (n=194)	csPCa (n=47)	Non csPCa (n=147)	P-value (U statistic) *
Age (years), median (IQR)	67 (62.2-72)	67 (63-72)	67 (62-71)	0.57 (U=3262)
PSA (ng/mL), median (IQR)	7.3 (5.6-9.3)	7.5 (5.9-9.1)	7.2 (5.5-9.3)	0.41 (U=3177)
Prostate Volume (mL), median (IQR)	52 (35-65.7)	37 (28.5-49.3)	57.6 (40.3-72.0)	<0.001 (U=5125)
PSA Density (PSAD) (ng/mL^2^), median (IQR)	0.142 (0.101-0.195)	0.212 (0.134-0.323)	0.132 (0.092-0.172)	<0.001 (U=1918)
Biopsy Histology Findings				
Benign	84	N/A	N/A	N/A
Grade 1 (Gleason 6)	63	N/A	N/A	N/A
Grade 2 (Gleason 3+4)	31	N/A	N/A	N/A
Grade 3 (Gleason 4+3)	9	N/A	N/A	N/A
Grade 4 (Gleason 8)	0	N/A	N/A	N/A
Grade 5 (Gleason 9/10)	7	N/A	N/A	N/A

Univariate analysis

On univariate logistic regression, higher PSAD (per 0.1 ng/mL2 increase) (OR 1.58; P=0.001) and smaller prostate volume (OR 0.96; p<0.001) were significantly associated with csPCa. Serum PSA demonstrated a borderline association (p=0.05). Age was not a significant predictor (p=0.41). 

Multivariate analysis

When age and PSAD (per 0.1 ng/mL2) were entered into a multivariate model, only PSAD remained an independent predictor of csPCa (OR 1.57, 95% CI 1.21-2.04, p=0.001). Age did not contribute significantly (p=0.47). The model had an overall classification accuracy of 76%. The results of the univariate and multivariate logistic regression are summarised in Table 2. 

**Table 2:** Univariate and Multivariate Logistic Regression Analysis for Prediction of Clinically Significant Prostate Cancer (csPCa). PSA: Prostate-Specific Antigen, PSAD: PSA Density

Variates	Univariate OR (95% CI)	P-value	Multivariate OR (95% CI)	P-value
Age (Years)	1.02 (0.97-1.07)	0.41	1.02 (0.97-1.07)	0.47
PSA (ng/mL)	1.06 (1.00-1.12)	0.05	-	-
Prostate Volume (mL)	0.96 (0.94-0.98)	<0.001	-	-
PSAD (ng/mL^2^) (x10)	1.58 (1.21-2.04)	<0.001	1.57 (1.21-2.03)	<0.001

Diagnostic performance by PSAD thresholds

**Figure 1:**
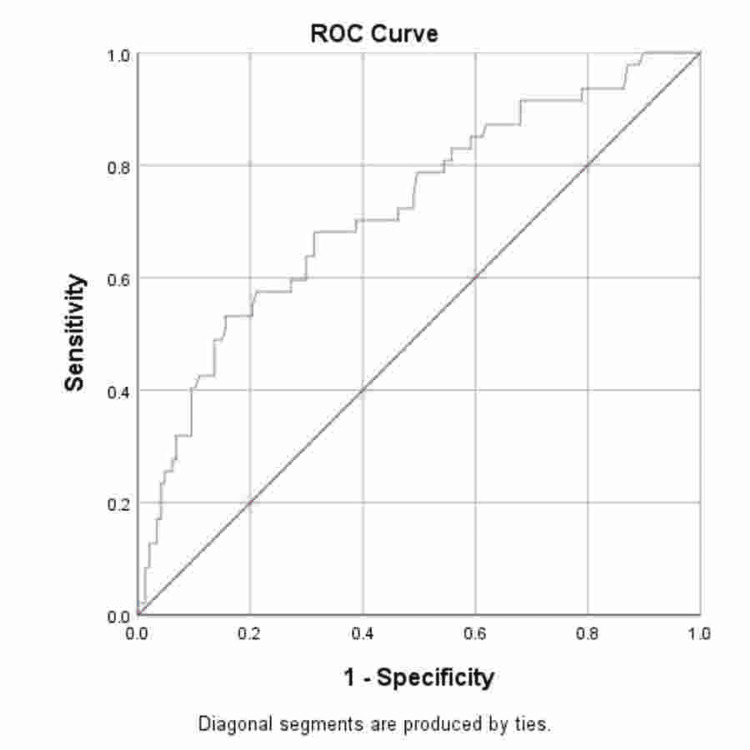
Receiver Operating Characteristic (ROC) Curve for Prostate-Specific Antigen Density (PSAD) in Predicting Clinically Significant Prostate Cancer (csPCa).

The ROC analysis demonstrated an AUC of 0.72 (95% CI 0.64-0.81, p<0.001) for PSAD in predicting csPCa. The ROC curve is shown in Figure 1. 

Diagnostic performance at various PSAD cut-offs is summarised in Table 3. At a PSAD of ≥ 0.10 ng/mL2, the sensitivity and specificity were 0.92 (95% CI 0.83-1.00) and 0.29 (95% CI 0.21-0.37), respectively, with a PPV of 0.29 (95% CI 0.21-0.37) and an NPV of 0.91 (95% CI 0.83-0.99). At ≥ 0.15 ng/mL2, sensitivity decreased to 0.68 (95% CI 0.55-0.81) and specificity increased to 0.65 (95% CI 0.58-0.73); PPV and NPV were 0.39 (95% CI 0.28-0.49) and 0.87 (95% CI 0.80-0.93), respectively. At the cut-off of ≥ 0.20 ng/mL2, sensitivity further decreased to 0.53 (95% CI 0.39-0.67) while specificity rose to 0.84 (95% CI 0.78-0.90); PPV and NPV were 0.52 (95% CI 0.38-0.66) and 0.85 (95% CI 0.79-0.91), respectively.

**Table 3:** Diagnostic Performance of Prostate-Specific Antigen Density (PSAD) Cut-Offs for Detecting Clinically Significant Prostate Cancer (csPCa).

PSAD cut-off (ng/mL^2^)	Sensitivity (95% CI)	Specificity (95% CI)	Positive Predictive Value (95% CI)	Negative Predictive Value (95% CI)
≥0.10	0.92 (0.83-1.00)	0.29 (0.21-0.36)	0.29 (0.21-0.37)	0.91 (0.83-0.99)
≥0.15	0.68 (0.55-0.81)	0.65 (0.58-0.73)	0.39 (0.28-0.49)	0.87 (0.80-0.93)
≥0.20	0.53 (0.39-0.67)	0.84 (0.78-0.90)	0.52 (0.38-0.66)	0.85 (0.79-0.91)

## Discussion

In our cohort, csPCa was identified in 47 (24.2%) of patients with PI-RADS 3 lesions, while 147 (75.8%) had non-csPCa or benign findings. A previous systematic review has found a detection rate of 18.5% in PI-RADS 3 patients [13]. This reinforces the diagnostic uncertainty associated with PI-RADS 3 lesions and the importance of refining other parameters, such as PSAD, to help risk-stratify. When examining baseline characteristics in our PI-RADS 3 population, we found that median prostate volume (37ml) was significantly lower and PSAD (0.21) was significantly higher in men with csPCa compared to men in the non-csPCa group (P<0.001). Age and PSA were not significant predictors of csPCa. Similar findings were observed in a study by Drevik et al. in which PSAD and prostate volume were significant (P<0.001), whilst age, PSA, and race were not [14]. 

In our study, the ROC analysis yielded an AUC of 0.72, indicating moderate discrimination and supporting the potential utility of this variable in clinical decision-making. Our multivariate analysis showed PSAD remained as the only independent predictor of csPCa. It demonstrated an adjusted OR of 1.57 (CI 1.21-2.03, p<0.001), indicating that a 0.1-unit increase was associated with a 57% increased likelihood of clinically significant cancer. These findings highlight the role of PSAD as a robust marker of independently predicting csPCa [15]. 

The sensitivity levels for our PSAD cut-offs of 0.10, 0.15, and 0.20 (ng/mL2) were 0.92, 0.68, and 0.53, respectively, demonstrating that the higher the PSAD threshold, the greater the risk of missing csPCa. This finding is consistent with a study by Knaapila et al., which found that, using the same cut-off values, sensitivities were 0.93, 0.78, and 0.56, respectively [16]. The inverse is true when we look at the specificity for the same dataset. For the same PSAD cut-off values, the specificity in our data was 0.29, 0.65, and 0.84, demonstrating that with a higher PSAD cut-off, clinicians are more certain in detecting csPCa. Our data shows that the NPV was 0.91, 0.87, and 0.85 and the PPV was 0.29, 0.39, and 0.52 for each subsequent PSAD threshold. In comparison, Nguyen et al. found the NPV for the same PSAD density cut-offs was 0.87, 0.85, and 0.80, and the PPV was 0.27, 0.40, and 0.32 [17]. We can infer from this that across all PSAD thresholds, the NPV levels remained high, indicating it is a good way to rule out the presence of csPCa confidently. On the other hand, our data showed an upward trend in PPV with increasing PSAD, suggesting that higher PSAD is associated with a greater likelihood of csPCa. However, this same trend is not observed in the study by Nguyen et al. [17].

When looking at our data and considering data from other studies, a cut-off value of 0.15 ng/mL2 is the optimal balance. A cut-off value of 0.15ng/mL2 has a sensitivity of 0.68, specificity of 0.65, PPV of 0.39, and NPV of 0.87. Venderink et al. found that with a PSAD cut-off> 0.15 ng/mL2, 42% of patients avoid harm from biopsy, whilst 6% of patients with csPCa will be missed [18]. The same study found that by lowering the cut-off to 0.12ng/mL2, no csPCa would have been missed, but only 26% would have avoided biopsy. Clinically, these findings highlight the importance of maintaining a delicate balance between avoiding unnecessary biopsies and ensuring that clinically significant cancers are not overlooked. Therefore, it remains crucial that csPCa is not missed, given the adverse outcomes associated with delayed or missed diagnosis. 

Strengths and limitations 

There are particular strengths of our study. We have exclusively focused on a cohort of patients with a PI-RADS 3 score with no prior history of prostate cancer, which is associated with diagnostic uncertainty. Outcomes have been defined using histopathological confirmation, thereby eliminating subjectivity related to imaging-based endpoints. Our quantitative approach and use of logistic regression analyses reinforces the statistical robustness of our findings. Furthermore, we have assessed PSAD at various cut-off points to enable direct comparison between different categories.

Our study is not without limitations. The analysis was a retrospective study conducted at a single centre in North Wales, which may affect the generalisability of the results to the broader population. As all the data were obtained from one institution and analysed retrospectively, there is an inherent risk of selection bias. Furthermore, we did not account for other patient variables, such as a positive family history, digital rectal examination findings, or race, which could influence the risk stratification of PI-RADS 3 patients for further management. We did not collect data on the patients’ clinical stage. Moreover, we did not include granular information regarding MRI acquisition protocols. Variability in imaging techniques or quality may influence the consistency of PI-RADS scoring and its diagnostic accuracy.

## Conclusions

This study assessed the ability of PSAD in identifying csPCa with PI-RADS 3 lesions at various thresholds. Our findings demonstrate that PSAD is the most influential independent predictor of clinically significant disease. A cut-off value of 0.15 ng/mL2 offers an optimal compromise between sensitivity and specificity for clinical risk stratification. While higher thresholds improve specificity at the expense of sensitivity, lower thresholds increase cancer detection but may lead to unnecessary biopsies. Further large-scale, multicentre, prospective studies are needed to confirm and refine these results.
